# The impact of ABCB1 and CES1 polymorphisms on the safety of dabigatran in patients with non-valvular atrial fibrillation

**DOI:** 10.1186/s12872-022-02910-4

**Published:** 2022-11-11

**Authors:** Zhu Zhu, Chenyue Qian, Cunjing Su, Hong Tao, Jiaojiao Mao, Zhening Guo, Xinyi Zhu, Jie Pan

**Affiliations:** 1 Department of Pharmacy, The Second Affiliated Hospital of Soochow University, 215004 Suzhou, Jiangsu China; 2 Department of cardiology, The Second Affiliated Hospital of Soochow University, 215004 Suzhou, Jiangsu China

**Keywords:** Dabigatran, ABCB1, CES1, Single nucleotide polymorphisms

## Abstract

**Background:**

This study aimed to analyze associations between genetic variants and plasma concentrations along with clinical outcomes in dabigatran in patients with non-valvular atrial fibrillation (NVAF).

**Methods:**

We conducted a prospective study and enrolled NVAF patients treated with dabigatran in the real world. A total of 86 patients treated with 110 mg DE twice daily were recruited for this study. Blood samples were obtained from each patient and used for genotyping and determination of plasma dabigatran concentration. All bleeding and thromboembolic complications were recorded during the 1.5 years of follow-up.

**Results:**

Eighty-three patients provided samples at the trough plasma level of dabigatran, and 58 patients provided samples at the peak plasma level of dabigatran. There was a significant association between the CES1 SNP rs8192935 and trough plasma concentrations of dabigatran (*P* = 0.013). Our results showed that the CES1 SNP rs8192935 significantly influenced dabigatran trough concentrations in the Chinese population, and carriers of the G allele had increased trough plasma concentrations of dabigatran compared to noncarriers. The ABCB1 SNP c.2482-2236G > A (rs4148738) was associated with major bleeding events in the addictive model (*P* = 0.046, OR = 3.29) and dominant model (*P* = 0.040, OR = 8.17). Additionally, the ABCB1 SNP c.3435 C > T (rs1045642) was associated with the incidence of major bleeding events in the addictive model (*P* = 0.043, OR = 3.34) and dominant model (*P* = 0.046, OR = 7.77). However, no significant associations were found between all the SNPs and the incidence of minor bleeding events.

**Conclusion:**

Our results indicated that the CES1 polymorphism rs8192935 was associated with trough plasma concentrations of dabigatran. Carriers of the G allele had increased trough plasma concentrations of dabigatran compared to noncarriers. The ABCB1 polymorphisms rs4148738 and rs1045642 were associated with an increased risk for major bleeding events for the first time in a Chinese population.

## Background

Non-valvular atrial fibrillation (NVAF) is the most common cardiac arrhythmia and is associated with an increased risk of ischemic stroke globally [1, 2]. Dabigatran etexilate (DE) is a novel non-vitamin K antagonist oral anticoagulant (NOAC) that is widely used in NVAF patients to prevent the formation of thrombus [3, 4]. DE was given in fixed-dose treatments without coagulation monitoring, which was shown to be more effective and safer for patients than vitamin K antagonists [3].

The effect of genetic factors on the pharmacokinetics of active dabigatran metabolites has been investigated in several studies [5–10]. Furthermore, dabigatran was also observed to cause clinical bleeding, which is closely associated with genetic polymorphisms [6, 7, 10]. The major genetic determinants involved in the metabolism of DE are the carboxylesterase 1 (CES1) and ATP binding cassette subfamily B member 1 (ABCB1) genes [11–13]. Single nucleotide polymorphisms (SNPs) of CES1 and ABCB1 have shown a significant effect on dabigatran dosing variations and their safety parameters.

However, few studies have examined Asian populations that differ in the risk of bleeding and genetic polymorphisms under anticoagulant therapy conditions, and the genetic impact on dabigatran PK/PD in Chinese or Asian populations remains unclear [12]. The only study conducted at Zhongshan Hospital indicated the effect of genetic associations of dabigatran along with the bleeding risk for individuals, but it failed to elucidate major bleeding and thromboembolic events of this variability due to the small number of patients enrolled in the study [6].

To date, very few pharmacogenetic studies on dabigatran in Asian patients have been published, and the evidence level of these associations is low. Therefore, the purpose of our study was to identify the impact of gene polymorphisms on the plasma concentration and clinical events of dabigatran in Chinese patients treated with DE for NVAF.

## Methods

### Study population

This was a prospective study performed in the atrial fibrillation (AF) clinic of the Second Affiliated Hospital of Soochow University in China between January 2018 and June 2019. A total of 86 patients treated with 110 mg DE twice daily were recruited for this study. All patients included in our study had received dabigatran for at least 18 continuous months and visited the clinic at least once a month for long-term follow-up. This study was approved by the Ethics Review Board of the Second Affiliated Hospital of Soochow University (approval number JD-LK-2018-069-01) and was performed in accordance with the Declaration of Helsinki. All patients signed informed consent prior to this study.

The inclusion criteria were [14]: (1) diagnosed with NVAF; (2) receiving anticoagulant therapy with oral DE for a minimum of eighteen months; and (3) provided at least one valid blood sample for SNP detection. We excluded patients who were pregnant, younger than 18 years of age, suffered severe vascular heart disease, severe liver damage or cancer, had prior kidney transplant or long-term dialysis, or were unable to fulfill the follow-up visits. The severity of liver disease was assessed and graded as Child-Pugh grade A, B or C. Severe liver damage was defined as Child-Pugh grade B to C [15]. Baseline characteristics were collected for all enrolled patients (shown in Table 1). All bleeding and thromboembolic complications were recorded during the 1.5 years of follow-up. Major bleeding was defined as a reduction in the hemoglobin level of at least 20 g/L, transfusion of at least 2 U blood, or symptomatic bleeding in a critical area or organ. All other bleeding was considered minor [14, 16]. Any bleeding events were composed of major and minor bleeding events.

**Table 1:** Demographics and clinical characteristics of dabigatran-treated patients enrolled in this study

Variables	Characteristics of the study population (n = 86)	Characteristics of the study population providing trough samples(n = 83)	Characteristics of the study population providing peak samples(n = 58)
Male, n(%)	49(56.98)	48(57.83)	31(53.44)
Age(years), mean(SD)	72.15 ± 9.17	72.12 ± 9.16	72.07 ± 9.27
BMI(kg/m2), mean(SD)	25.01 ± 3.76	25.15 ± 3.75	24.71 ± 4.08
Creatinine clearance, mL/min	70.44 ± 26.81	71.87 ± 26.66	69.82 ± 25.63
CHA2DS2-VASc, mean(SD)	3.41 ± 1.69	3.36 ± 1.63	3.33 ± 1.74
HAS-BLED, mean(SD)	1.15 ± 0.88	1.16 ± 0.89	1.02 ± 0.74
AST(U/L), mean(SD)	22.84 ± 11.91	22.82 ± 12.09	22.86 ± 11.76
ALT(U/L), mean(SD)	23.14 ± 16.55	22.84 ± 16.58	21.47 ± 16.52
History of hypertension, n(%)	62(72.09)	60(72.29)	41(70.69)
History of stroke, n(%)	14(16.28)	13(15.66)	7(12.07)
History of diabetes mellitus, n(%)	23(26.74)	22(26.51)	14(24.14)
Aspirin use, n(%)	3(3.49)	3(3.61)	1(1.72)
Clopidogrel use, n(%)	3(3.49)	3(3.61)	1(1.72)
Amiodarone use, n(%)	4(4.65)	4(4.82)	2(3.44)
Trough concentration(ng/mL), mean(SD)	51.32 ± 38.63 (n = 83)		
Peak concentration(ng/mL), mean(SD)	101.12 ± 56.3 (n = 58)		
Any ischemic event, n(%)	9(10.47)	9(10.84)	3(5.17)
Any bleeding, n(%)	17(19.77)	15(18.07)	14(24.17)
Major bleeding, n(%)	4(4.65)	4(4.82)	3(5.17)
Minor bleeding, n(%)	13(15.12)	11(13.25)	11(18.97)

### Selection of SNPs and genotyping

A total of 86 patients treated with 110 mg DE twice daily were recruited for this study. Peripheral blood samples (3 mL) were collected in EDTA anticoagulant tubes for each patient. Genomic DNA was extracted using a Genomic DNA Purification Kit according to standard protocols. Genotyping of each polymorphism in the ABCB1 and CES1 genes was carried out by the MassARRAY® MALDI-TOF System.

Four SNPs in 2 genes (ABCB1 and CES1) were selected from the National Center for Biotechnology Information (NCBI) database (Table 2). All of the SNPs satisfied the following criteria: (1) SNPs that are associated with cardiac arrhythmia occurrence or development according to the results of existing research [17–22]; (2) SNPs that may influence the function or expression of ABCB1 and CES1 by NCBI; and (3) minor allele frequency (MAF) ≥ 5% in the Chinese Han population.

**Table 2:** Allele frequencies by loci for ABCB1 and CES1 in the subjects

Gene	SNP	Genotype	N(%)	Minor allele	MAF(%)	HWE, p value
**ABCB1**	**rs4148738**	CC	18(16.1)	C	39.7	0.9
		CT	53(47.3)			
		TT	41(36.6)			
	**rs1045642**	AA	18(16.1)	A	37.9	0.45
		AG	49(43.8)			
		GG	45(40.2)			
**CES1**	**rs2244613**	GG	43(38.4)	T	40.2	0.25
		GT	48(42.8)			
		TT	21(18.8)			
	**rs8192935**	AA	65(58.0)	G	22.8	0.33
		AG	43(38.4)			
		GG	4(3.6)			

Abbreviations: SNP, single nucleotide polymorphism; MAF, minor allele frequency; HWE, Hardy-Weinberg equilibrium

### Measurement of dabigatran plasma concentration

A total of 3 ml of peripheral blood was collected from each patient, and those collected 12 h after the previous dose were considered at the trough plasma level of dabigatran, while those obtained 2 h after the dose were considered at the peak level. Blood samples containing dabigatran were obtained from whole blood by centrifugation at 3000 rpm at 4 °C for 5 min and stored at -80 °C for further detection. The plasma concentration of DE was detected by UPLC–MS/MS as described previously [14].

### Statistical analysis

The chi-squared test was used to evaluate the Hardy-Weinberg equilibrium or allele frequencies of all SNPs [23, 24]. Unconditional logistic regression was conducted to calculate the adjusted odds ratio (OR) and 95% confidence intervals (95% CI). The association analysis was tested using logistic regression analysis with PLINK version 1.07 [25] (PLINK is a free, open-source whole genome association analysis toolset) under additive, dominant, and recessive models. The test of significance was set at two-tailed *P* = 0.05. The Mann-Whitney U test and the Kruskal-Wallis H test were used to evaluate the associations between single nucleotide polymorphisms and plasma concentrations of dabigatran. The Mann-Whitney U test was used to compare two independent samples, and the Kruskal-Wallis H test was used to compare multiple independent samples.

## Results

A total of 86 patients treated with 110 mg DE twice daily were recruited for this study. All subjects were Chinese and had a mean (SD) age of 72.15 ± 9.17 at the time of recruitment. Four SNPs were genotyped in these subjects, and all SNPs conformed to Hardy-Weinberg equilibrium (P > 0 05) (shown in Table 2). Eighty-three patients provided samples at the trough plasma level of dabigatran, and 58 patients provided samples at the peak plasma level of dabigatran. Peak and trough plasma concentrations of different genotypes are shown in Table 3.

**Table 3:** Association between single nucleotide polymorphisms and peak and trough plasma concentrations of dabigatran

Gene	SNP	Genotype	Trough (ng/mL)		Peak (ng/mL)	
ABCB1	**rs4148738**	CC	N = 13	46.20 ± 31.10	N = 10	82.30 ± 58.66
		CT	N = 45	48.80 ± 42.93	N = 26	97.33 ± 60.65
		TT	N = 25	58.52 ± 34.93	N = 22	114.16 ± 50.35
		P value		0.535		0.310
	**rs2032582**	AT	N = 17	43.12 ± 34.16	N = 6	87.89 ± 54.86
		CC	N = 11	45.95 ± 31.91	N = 9	109.01 ± 41.55
		AA	N = 13	46.20 ± 31.10	N = 10	82.30 ± 58.66
		AC	N = 28	52.26 ± 47.74	N = 20	100.16 ± 63.34
		TT	N = 4	65.23 ± 34.14	N = 3	154.06 ± 74.07
		CT	N = 10	69.66 ± 37.17	N = 10	106.83 ± 50.45
		P value		0.568		0.520
	**rs1045642**	AA	N = 13	43.01 ± 32.90	N = 11	75.55 ± 59.19
		AG	N = 41	51.24 ± 44.78	N = 23	99.44 ± 51.85
		GG	N = 29	55.17 ± 32.39	N = 24	114.45 ± 58.29
		P value		0.650		0.169
CES1	**rs2244613**	GG	N = 32	44.21 ± 30.20	N = 20	89.16 ± 57.00
		GT	N = 37	58.03 ± 45.72	N = 28	113.09 ± 58.86
		TT	N = 14	49.86 ± 36.21	N = 10	91.54 ± 48.04
		P value		0.338		0.304
	**rs8192935**	AA	N = 45	42.73 ± 29.04	N = 31	89.58 ± 54.35
		AG	N = 36	58.66 ± 45.46	N = 25	112.23 ± 58.03
		GG	N = 2	112.69 ± 40.63	N = 2	141.08 ± 63.29
		P value		0.013^*^		0.202

Data are expressed as the mean ± standard deviation

*P<0.05

There was a significant association between the CES1 SNP rs8192935 and trough plasma concentrations of dabigatran (*P* = 0.013). For the trough levels, carriers of the G allele had increased trough plasma concentrations of dabigatran compared to the noncarriers. No significant association was observed between other SNPs and the plasma concentration of dabigatran (Table 3).

In an exploratory analysis, ischemic and bleeding events related to patients treated with DE were compared among different genotypes of ABCB1 and CES1 (Table 4). Clinical outcomes, including ischemic events and bleeding events, were recorded throughout the 1.5-year follow-up. All bleeding events were composed of major and minor bleeding events.

**Table 4:** Association between single nucleotide polymorphisms and ischemic events and bleeding in all subjects

Gene	SNP	Mod	Stroke/SEE		Any Bleeding		Major bleeding		Minor bleeding	
			OR (95%CI)	P	OR (95%CI)	P	OR (95%CI)	P	OR (95%CI)	P
ABCB1	rs4148738	Add	1.52 (0.62, 3.70)	0.359	2.29 (1.05, 5.01)	0.038^*^	3.29 (1.02, 10.59)	0.046^*^	1.59 (0.73, 3.44)	0.240
		Dom	1.50 (0.63, 3.60)	0.360	2.30 (1.06, 4.96)	0.035^*^	8.17 (1.10, 60.88)	0.040^*^	1.61 (0.74, 3.49)	0.225
		Rec	1.65 (0.65, 4.15)	0.290	2.19 (1.00, 4.76)	0.048^*^	2.55 (0.93, 7.03)	0.070	1.56 (0.71, 3.42)	0.266
	rs1045642	Add	1.45 (0.60, 3.49)	0.404	2.31 (1.06, 5.03)	0.034^*^	3.34 (1.04, 10.74)	0.043^*^	1.61 (0.74, 3.50)	0.230
		Dom	1.47 (0.61, 3.52)	0.388	2.27 (1.06, 4.87)	0.034^*^	7.77 (1.03, 58.39)	0.046^*^	1.62 (0.74, 3.49)	0.225
		Rec	1.52 (0.62, 3.74)	0.362	2.20 (1.00, 4.82)	0.049^*^	2.50 (0.89, 7.00)	0.083	1.57 (0.72, 3.46)	0.260
CES1	rs2244613	Add	1.55 (0.63, 3.79)	0.339	2.30 (1.05, 5.04)	0.038^*^	2.62 (0.95, 7.21)	0.062	1.61 (0.74, 3.50)	0.225
		Dom	1.50 (0.63, 3.60)	0.360	2.28 (1.04, 5.00)	0.038^*^	2.61 (0.94, 7.24)	0.066	1.62 (0.74, 3.51)	0.227
		Rec	1.54 (0.63, 3.77)	0.342	2.33 (1.06, 5.14)	0.035^*^	2.67 (0.97, 7.33)	0.058	1.63 (0.75, 3.55)	0.216
	rs8192935	Add	1.67 (0.65, 4.29)	0.290	2.26 (1.03, 4.96)	0.042^*^	2.50 (0.88, 7.05)	0.084	1.61 (0.74, 3.53)	0.232
		Dom	1.67 (0.65, 4.31)	0.290	2.31 (1.04, 5.10)	0.039^*^	2.39 (0.83, 6.83)	0.105	1.67 (0.75, 3.74)	0.209
		Rec	1.54 (0.63, 3.75)	0.348	2.42 (1.08, 5.42)	0.031^*^	2.65 (0.96, 7.34)	0.060	1.72 (0.77, 3.79)	0.180

OR: odds ratio; 95%CI: 95% confidence interval; SEE: systemic embolic events; ADD: additive model; DOM: dominant model; REC: recessive model; additive model: comparing the minor allele with major allele subjects; dominant model: comparing carriers of the minor allele with the major homozygous subjects; recessive model: comparing carriers of the major allele with the minor homozygous subjects

*P<0.05

Overall, the incidence of ischemic events (stroke/SEE) in the included population was 10.47%. The incidence of ischemic events did not vary considerably among genotypes of ABCB1 or CES1. The incidence of any bleeding event in the included population was 19.77%. There was a significant association between all the SNPs and the incidence of any bleeding event (Table 4). Further analysis showed that there was a significant association between the ABCB1 SNP c.2482-2236G > A (rs4148738) and major bleeding events in the addictive model (*P* = 0.046, OR = 3.29) and dominant model (*P* = 0.040, OR = 8.17). Additionally, the ABCB1 SNP c.3435 C > T (rs1045642) was associated with the incidence of major bleeding events in the addictive model (*P* = 0.043, OR = 3.34) and dominant model (*P* = 0.046, OR = 7.77). However, no significant associations were found between all the SNPs and the incidence of minor bleeding events.

## Discussion

The polymorphisms of both ABCB1 and CES1 genes may lead to a significant association with pharmacokinetic variations and clinical outcomes of dabigatran [8, 20, 26]. In this study, we found that the CES1 SNP rs8192935 was associated with the plasma concentrations of dabigatran (*P* = 0.013). For the trough levels, carriers of the G allele had increased trough plasma concentrations of dabigatran compared to the noncarriers. The ABCB1 SNPs rs4148738 and rs1045642 were associated with a higher risk for major bleeding in patients receiving dabigatran treatment. However, no significant difference was observed in ischemic events or minor bleeding events among variant genotypes of the ABCB1 SNPs and the CES1 SNPs.

CES1 is responsible for the biotransformation of dabigatran etexilate into the active metabolite [13]. Genome-wide association analysis identified that the CES1 SNP rs2244613 is associated with trough dabigatran blood concentration, while rs8192935 and rs4148738 are modestly associated with peak dabigatran blood concentration [7]. A Chinese patient case report showed that the impacts on dabigatran concentrations related to rs2244613 and rs8192935 may be greater than previously postulated, especially in Asians [5]. Thus, more studies should be conducted in the Chinese population.

Our results showed that the CES1 SNP rs8192935 significantly influenced dabigatran trough concentrations in the Chinese population, and carriers of the G allele had increased trough plasma concentrations of dabigatran compared to noncarriers, especially for the patients with GG genotype showed higher trough concentrations (*P* = 0.013). This result is in accordance with a previous study in Italy and China [6, 13]. To the best of our knowledge, only one study conducted in China indicated the effect of genetic associations of dabigatran along with the bleeding risk for individuals, but it failed to elucidate major bleeding and thromboembolic events of this variability due to the small number of patients enrolled in the study [6]. Our results reverified the importance of the CES1 SNP rs8192935 with larger enrolled patient numbers and included ischemic events, major bleeding events and minor bleeding events in further studies. However, the CES1 SNP rs8192935 showed no significant difference in ischemic events or minor bleeding events at the 1.5-year follow-up.

Dabigatran etexilate is a substrate of P-glycoprotein encoded by the ABCB1 gene. ABCB1 variants are potential factors affecting thromboembolic events in dabigatran users and bleeding events [27]. Earlier studies have found associations between genetic variability and plasma levels of direct oral anticoagulants [17, 28]. ABCB1 SNP 1,045,642 is associated with a reduced risk for thromboembolic outcomes, while rs4148738 is associated with a lower risk for bleeding events in apixaban users. For the ABCB1 polymorphisms rs4148738 and rs1045642, no significant association was previously found with dabigatran PK/PD in the Chinese population [6].

It can be found that the trough and peak concentrations of the ABCB1 rs4148738 TT genotype are significantly higher than those of other genotypes (Table 3). Similarly, the trough and peak concentrations of the ABCB1 rs1045642 GG genotype are also significantly higher than those of other genotypes, and the trend is very obvious. However, no statistical difference was shown due to the limited sample size. The genetic polymorphism of CES1 rs8192935 was associated with any bleeding event. However, due to the limited sample size and the large coefficient of variation of blood drug concentration, there was no correlation between CES1 rs8192935 polymorphisms and peak-to-trough concentrations.

In our study, no significant associations were found between the plasma concentrations of dabigatran and ABCB1 polymorphisms. The results are in accordance with a previous study in China. However, the ABCB1 SNPs rs4148738 and rs1045642 were detected to have significant associations with a higher risk for major bleeding in patients receiving dabigatran treatment in the Chinese population for the first time. However, they showed no significant difference in ischemic events or minor bleeding events at the 1.5-year follow-up. Since our research has a longer follow-up and we have a strict selection criterion for the patients, further research should be conducted in the future.

Our study also has some limitations. First, our study only included specific populations (excluded patients with severe hepatic and renal insufficiency, cancer patients), so our findings may not be applicable to other broader clinical populations. Secondly, the samples we included were limited, and each patient only provided 1–2 dabigatran plasma concentration data, which made it impossible to accurately draw the PK curve and provide more data support for the clinic. Moreover, due to the small sample size, there may be some statistical errors in the correlation study of gene polymorphisms with trough concentrations and clinical outcomes. Finally, this study is a single-center study, which may introduce some selection bias.

## Conclusion

In our real-world study, our results indicated that the CES1 polymorphism rs8192935 was associated with trough plasma concentrations of dabigatran. Carriers of the G allele had increased trough plasma concentrations of dabigatran compared to noncarriers. The ABCB1 polymorphisms rs4148738 and rs1045642 were associated with an increased risk for major bleeding events. These genetic associations could be useful for predicting the effect of dabigatran along with the bleeding risk for individuals, and further studies with larger sample sizes are needed to verify the findings.

## Data Availability

The datasets used and analyzed during the current study are available from the corresponding author on reasonable request.

## Abbreviations

NVAF: Non-valvular atrial fibrillation
NOAC: non-vitamin K antagonist oral anticoagulant
DE: Dabigatran etexilate
CES1: carboxylesterase 1
ABCB1: ATP binding cassette subfamily B member 1
SNP: Single nucleotide polymorphism.
